# Eyelid squinting improves near vision in against-the-rule and distance vision in with-the-rule astigmatism in pseudophakic eyes: an eye model experimental study

**DOI:** 10.1186/s12886-019-1297-5

**Published:** 2020-01-02

**Authors:** Jay Won Rhim, Youngsub Eom, Seo Yeon Park, Su-Yeon Kang, Jong Suk Song, Hyo Myung Kim

**Affiliations:** 1 Miso Eye Clinic, Seoul, South Korea; 2 Department of Ophthalmology, Korea University College of Medicine, Seoul, South Korea; 3 Department of Ophthalmology, Korea University Ansan Hospital, 123, Jeokgeum-ro, Danwon-gu, Ansan-si, Gyeonggi-do 15355 South Korea

**Keywords:** Pseudoaccommodation, Eyelid, Astigmatism, Against-the-rule, With-the-rule, Pseudophakia

## Abstract

**Background:**

To elucidate whether eyelid squinting improves near and distance vision in against-the-rule (ATR) and with-the-rule (WTR) simple myopic astigmatism in pseudophakic eyes.

**Methods:**

A refraction-model eye was mounted on a wavefront analyzer. The eyelid fissure was simulated using a slit placed horizontally in front of the model eye. Four different refractive statuses [− 1.50 diopters (D) and − 3.00 D of both WTR and ATR simple myopic astigmatism] were set using cylindrical lenses. For each refractive status (emmetropia, − 1.50 D WTR, − 1.50 D ATR, − 3.00 D WTR, and − 3.00 D ATR astigmatism), wavefront aberrations were measured, both with and without the slit, 40 times each.

**Results:**

The 2 mm horizontal slit caused a hyperopic focus shift (+ 6.69 μm) in − 1.50 D WTR astigmatism, whereas, in − 1.50 D ATR astigmatism, it caused a myopic focus shift (− 2.01 μm). The astigmatism was decreased in the ATR astigmatism groups and increased in the emmetropia and WTR astigmatism groups, respectively. Total aberrations were decreased in the emmetropia and WTR astigmatism groups and increased in the ATR astigmatism groups. When the reference plane was set to the near plane, total aberrations were decreased in the ATR astigmatism groups.

**Conclusion:**

As the horizontal slit was placed in front of the model eye, the focus moves nearer in ATR astigmatism and farther in WTR astigmatism. These effects of eyelid cause improvement of near vision of pseudophakic eyes with ATR astigmatism.

## Background

To date, most clinical trials that address the topic of vision have been performed using visual acuity (VA) at distance and near as the standard outcome parameters, because refractive state as well as the effects of aberration on VA are of utmost interest to ophthalmologists [1]. Both distant and near vision are affected by pseudoaccommodation, which is defined as an increase in the depth of focus by a means other than true accommodation; this is achieved by changing the focal length of the optical system [2]. Factors influencing pseudoaccommodation include mild myopic astigmatism, pupil size, and corneal multifocality [3]. However, to the best of our knowledge, the effects of the eyelid fissure as a factor of pseudoaccommodation have not yet been considered.

Many studies have addressed the pseudoaccommodation effect of astigmatism, and near vision is known to be impacted by the magnitude and meridian of astigmatism, [3–14] pupil size, [15–20] and the shape of the optotype [21–23]. Investigations performing a visual quality comparison between with-the-rule (WTR) and against-the-rule (ATR) astigmatism have shown discordant results, likely because of differences in their methods, outcome measures, and influencing factors. For this reason, precise conclusions cannot be drawn as to whether WTR or ATR astigmatism is better for near vision [3–14, 24]. Nevertheless, many studies have shown that near vision tends to be better in low-myopic ATR astigmatism than in WTR astigmatism [3–7]. The definitive reason for this finding is unclear; indeed, it may simply be an artifact of the use of the Latin alphabet, in that the letters have a greater vertical than horizontal component [2].

Since the eyelids are in the vicinity of the optical axis, they could easily and greatly affect an individual’s vision. Many people use squinting (narrowing the eyelids to create a pinhole effect) to improve VA [25]. However, it is unclear how and for whom exactly this action improves near vision. When one looks downwards to see close-up objects, the upper eyelid descends and the eyelid fissure narrows [26]. People with presbyopia or ametropia often squint to see better. When the eyelids are closed via squinting to be smaller than the entrance pupil, they obstruct some part of the scattered light rays reaching the retina. Even though the eyelids could have a positive effect on VA, past studies of the eyelids in relation to vision have mainly focused on their detrimental effects [27–31]. Grey and Yap observed increased WTR astigmatism by use of an autorefractor when the eyelid fissure was deliberately narrowed [28]. Buehren et al. showed that natural forces applied by the eyelids for an hour are capable of altering corneal topography [29]. However, these clinical studies have many intersubject variations (e.g., eyelid tension, eyelid fissure size, accommodation, eyelashes, tear film), which were not controlled. To rule out the effects of eyelid pressure and to evaluate the pure optical change in eyes with narrowed eyelid fissures, this study measured wavefront aberrations of a refraction-model eye with and without the slit for each refractive status (i.e., emmetropia, − 1.50 diopters [D] WTR, − 1.50 D ATR, − 3.00 D WTR, and − 3.00 D ATR simple myopic astigmatism) at different distances.

## Methods

### Finite schematic eye model mounted on a wavefront aberrometer

A refraction-simulation model eye (Heine Optotechnik, Herrsching, Germany) was used as a finite schematic eye model. It contained a single achromatic lens with a focal length of 32 mm as well as a size-adjustable aperture stop behind the lens to simulate a pupil. The entrance pupil of the human eye is typically about 4 mm in diameter [32, 33] and changes between 2 mm and 8 mm with regard to the amount of ambient light present. In this study, we set the pupil size at both 4 mm and 6 mm.

The model eye was mounted on a wavefront aberrometer (WASCA; Carl Zeiss Meditec AG, Jena, Germany). Refraction and wavefront measurements were done through use of a point-source LASER targeted only at the center of the retina. An attenuation filter was placed in front of the aberrometer to control source light intensity.

### Simulation of squinting and refractive error

Squinting was simulated by placing 2 mm horizontal slit in front of the model eye, because pinhole size of 2 mm has a sufficient pinhole effect for optimal near vision without the effect of reduced retinal illuminance by small pinhole [34, 35]. To evaluate the effects of eyelid fissure on near vision with WTR and ATR astigmatism, lid fissure configuration needed to be simplified, although the human eyelid fissure is slightly curved and angled and eyelashes also have an influence on aberration measurement error.

Four different refractive error statuses (− 1.50 D and − 3.00 D of both WTR and ATR simple myopic astigmatism) were simulated using cylindrical lenses. Specifically, a + 1.50 D cylindrical lens was inserted to induce − 1.50 D astigmatism, while a + 3.00 D cylindrical lens was inserted to induce − 3.00 D astigmatism. The cylindrical lenses were rotated 90 degrees to simulate WTR or ATR astigmatism as necessary.

### Measurement of SEIDEL refraction and aberration

**Fig. 1:**
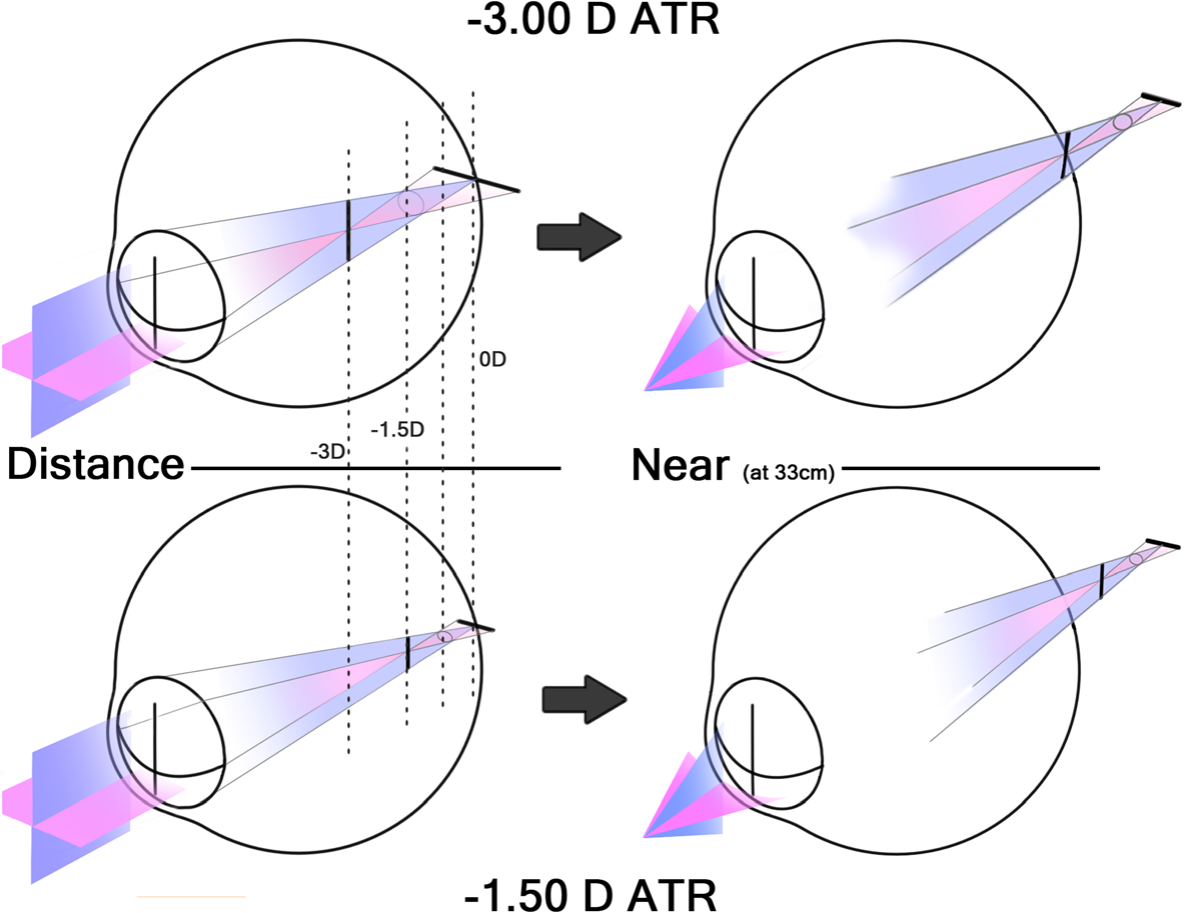
Four reference planes wherein focal lines and circle of least confusion coincide at the retina at a specific distance (left). As the object gets nearer, these planes move toward the retina (right)

At each refractive status (i.e., emmetropia, − 1.50 D WTR, − 1.50 D ATR, − 3.00 D WTR, and − 3.00 D ATR simple myopic astigmatism), Seidel refraction (e.g., spherical and cylindrical refractive error), Seidel aberrations (e.g., focus, astigmatism, coma, spherical aberration, and higher orders), and wavefront aberrations of Zernike polynomials were recorded with and without the slit, 40 times each. With different analysis diameters (simulating pupil sizes of 4 mm and 6 mm), wavefront measurements were conducted using the same method. To change distance (reference plane), a defocus method was used. Distances were set at 33 cm (− 3.00 D), 66 cm (− 1.50 D), 1.3 m (− 0.75 D), and 6 m (0 D), respectively, where vertical, horizontal focal lines, or a circle of least confusion at specific distances were present (Fig. 1).

### Vision chart simulation

The Complete Ophthalmic Analysis System (COAS) vision simulation program (included in the WASCA system) renders a Modulation Transfer Function of aberration data on the tumbling E chart. Vision chart information as seen through the eyes of each refractive status at different distances were obtained.

## Results

**Fig. 2:**
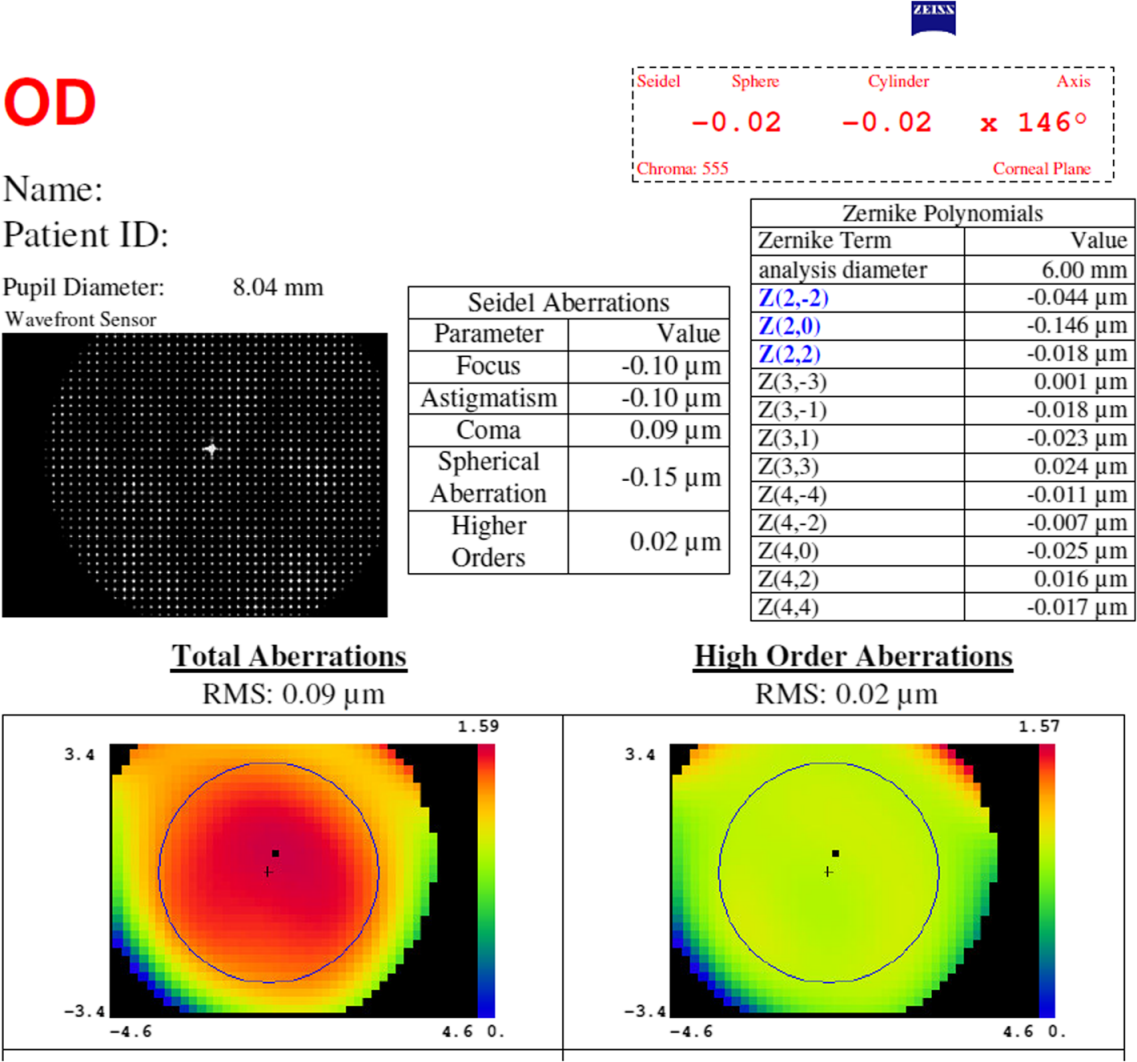
Wavefront aberrations of the model eye set to emmetropia, showing minimal aberration

Forty analyses of the emmetropia group without slit revealed a mean spherical refractive error of − 0.06 D, a mean cylindrical error of − 0.08 D, and a mean spherical aberration of − 0.15 μm. None of the aberration terms were greater than ±0.03 μm, except for defocus (Z_2_^0^), which was − 0.14 μm (Fig. 2).

Results with the analysis diameter set to 6 mm are shown in Table 1. As the 2 mm horizontal slit was placed in front of the model eye, the spherical power in the − 1.50 D WTR astigmatism revealed a hyperopic shift from − 0.28 D to + 1.21 D, whereas the spherical power in the − 1.50 D ATR astigmatism showed a myopic shift from − 0.29 D to − 0.74 D. Likewise, in the − 3.00 D WTR astigmatism, the spherical power revealed a hyperopic shift from − 0.31 D to + 0.46 D, whereas the spherical power in the − 3.00 D ATR astigmatism showed a myopic shift from − 0.25 D to − 0.60 D. Similarly, the focus term of the Seidel aberrations coefficient in the − 1.50 D WTR astigmatism revealed a hyperopic shift of + 6.69 μm, whereas the focus in the − 1.50 D ATR astigmatism showed a myopic shift of − 2.01 μm. In the same way, in the − 3.00 D WTR astigmatism, the focus revealed a hyperopic shift of + 3.43 μm, whereas the focus in the − 3.00 D ATR astigmatism showed a myopic shift of − 1.57 μm.

**Table 1:** Seidel refraction and Seidel aberrations coefficients with and without the horizontal slit, and the amount of change in the setting of an analysis pupil diameter of 6 mm and a slit size of 2 mm

	Emmetropia			− 1.50 D WTR			− 3.00 D WTR			− 1.50 D ATR			− 3.00 D ATR		
	No slit	Slit	Change	No slit	Slit	Change	No slit	Slit	Change	No slit	Slit	Change	No slit	Slit	Change
Spherical (D)	−0.11	0.14	0.25	−0.28	1.21	1.49	−0.31	0.46	0.77	−0.29	−0.74	−0.45	−0.25	−0.60	−0.35
Cylindrical (D) ^†^	−0.06	−0.47	−0.41	− 1.44	−3.55	−2.11	−2.88	−3.99	−1.11	− 1.48	−0.15	1.33	− 2.85	− 2.03	0.82
SEQ (D)	−0.14	− 0.10	0.05	−1.00	−0.57	0.44	−1.75	−1.54	0.22	−1.03	−0.82	0.22	−1.68	−1.62	0.06
Focus (μm)	−0.49	0.61	1.10	−1.25	5.44	6.69	−1.37	2.06	3.43	−1.32	−3.33	−2.01	−1.14	−2.71	−1.57
Astigmatism (μm)	−0.70	−2.11	−1.41	−6.49	−15.96	−9.47	−12.95	−17.94	−4.99	−6.66	−0.69	5.97	−12.81	−9.15	3.66
HOA (μm)	0.05	0.18	0.13	0.04	0.66	0.62	0.05	0.39	0.34	0.04	0.52	0.48	0.04	0.22	0.18
TA (μm)	0.32	0.30	−0.02	1.83	0.59	−1.24	3.51	1.16	−2.35	1.85	2.21	0.36	3.41	3.91	0.50
TA at 67 cm (μm)										1.82	0.62	−1.20	2.73	2.14	−0.59
TA at 33 cm (μm)										3.43	1.97	−1.46	3.59	0.57	−3.02

D = diopter; WTR = with-the-rule; ATR = against-the-rule; SEQ = spherical equivalent; HOA = high-order aberrations; TA = total aberrations

^†^ Cylinder axis is WTR in emmetropia, − 1.50 D WTR, and − 3.00 D WTR astigmatism and is ATR in − 1.50 D ATR and − 3.00 D ATR astigmatism

Placement of the 2 mm horizontal slit in front of the model eye induced an increase of astigmatism in the emmetropia, − 1.50 D WTR, and − 3.00 D WTR astigmatism (i.e., WTR cylindrical power was increased by − 0.41 D, − 2.11 D, and − 1.11 D, respectively), while there was a decrease of astigmatism in the − 1.50 D ATR and − 3.00 D ATR astigmatism (i.e., ATR cylindrical power was decreased by − 1.33 D and − 0.82 D, respectively) with such. Likewise, the astigmatism term of the Seidel aberrations coefficient was increased in the emmetropia, − 1.50 D WTR, and − 3.00 D WTR astigmatism and decreased in the − 1.50 D ATR and − 3.00 D ATR astigmatism (Table 1).

As the 2 mm horizontal slit was placed in front of the model eye, higher order aberrations were increased in all groups. Total aberrations were decreased in the emmetropia and the WTR astigmatism groups, while the ATR astigmatism groups demonstrated an increase in total aberrations. However, when the reference plane was adjusted for near distance of 67 cm and 33 cm, respectively, Total aberrations were decreased (specifically from 1.82 μm to 0.62 μm in the − 1.50 D ATR astigmatism and from 3.59 μm to 0.57 μm in the − 3.00 D ATR astigmatism) (Table 1).

Results of when the analysis diameter was set to 4 mm (pupil size: 4 mm) are shown in Table 2. Most of the changes occurred in the same direction but to a smaller degree as compared with the results of when the diameter was set to 6 mm.

**Table 2:** Seidel refraction and Seidel aberrations coefficients with and without the horizontal slit, and the amount of change in the setting of an analysis pupil diameter of 4 mm and a slit size of 2 mm

	Emmetropia			− 1.50 D WTR			−3.00 D WTR			−1.50 D ATR			−3.00 D ATR		
	No slit	Slit	Change	No slit	Slit	Change	No slit	Slit	Change	No slit	Slit	Change	No slit	Slit	Change
Spherical (D)	−0.17	−0.06	0.11	−0.32	0.36	0.68	−0.31	0.04	0.35	−0.56	−0.62	−0.06	−0.51	−0.53	−0.02
Cylindrical (D) ^†^	−0.17	−0.20	−0.03	−1.48	−2.03	−0.55	−2.81	−3.18	−0.37	−1.46	−1.12	0.34	−2.80	−2.50	0.30
SEQ (D)	−0.26	−0.16	0.10	−1.06	−0.66	0.41	−1.72	−1.55	0.17	−1.29	−1.18	0.11	−1.91	−1.78	0.13
Focus (μm)	−0.35	−0.12	0.23	−0.64	0.73	1.37	−0.63	0.09	0.72	−1.13	−1.28	−0.15	−1.01	−1.21	−0.20
Astigmatism (μm)	−0.33	−0.40	−0.07	−2.97	−4.05	−1.08	−5.62	−6.36	−0.74	−2.91	−2.23	0.68	−5.61	−5.01	0.60
HOA (μm)	0.02	0.15	0.13	0.04	0.18	0.14	0.04	0.12	0.08	0.04	0.14	0.10	0.04	0.08	0.04
TA (μm)	0.14	0.17	0.03	0.82	0.51	−0.31	1.54	1.09	−0.45	0.85	0.98	0.13	1.50	1.61	0.11
TA at 67 cm (μm)										0.81	0.49	−0.32	1.22	1.01	−0.21
TA at 33 cm (μm)										1.51	0.81	−0.70	1.61	0.52	−1.09

D = diopter; WTR = with-the-rule; ATR = against-the-rule; SEQ = spherical equivalent; HOA = high-order aberrations; TA = total aberrations

^†^ Cylinder axis is WTR in emmetropia, − 1.50 D WTR, and − 3.00 D WTR astigmatism and is ATR in − 1.50 D ATR and − 3.00 D ATR astigmatism

**Fig. 3:**
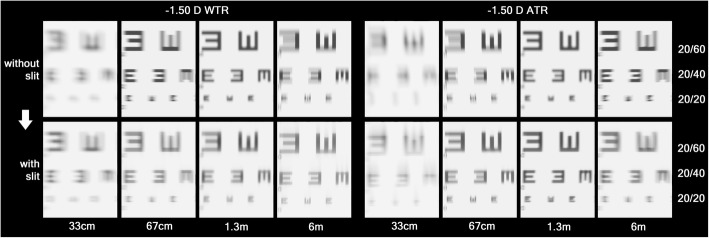
Simulated vision chart seen through the refractive statuses of − 1.50 D WTR and ATR simple myopic astigmatism (COAS vision simulation program). Without the slit, both groups could see best at a 1.3 m distance (at which the circle of least confusion coincides on the retina). With the slit, the distance at which the vertical focal lines lie on the retina showed better vision than before (6 m of the − 1.50 D WTR astigmatism and 67 cm of the − 1.50 D ATR astigmatism)

**Fig. 4:**
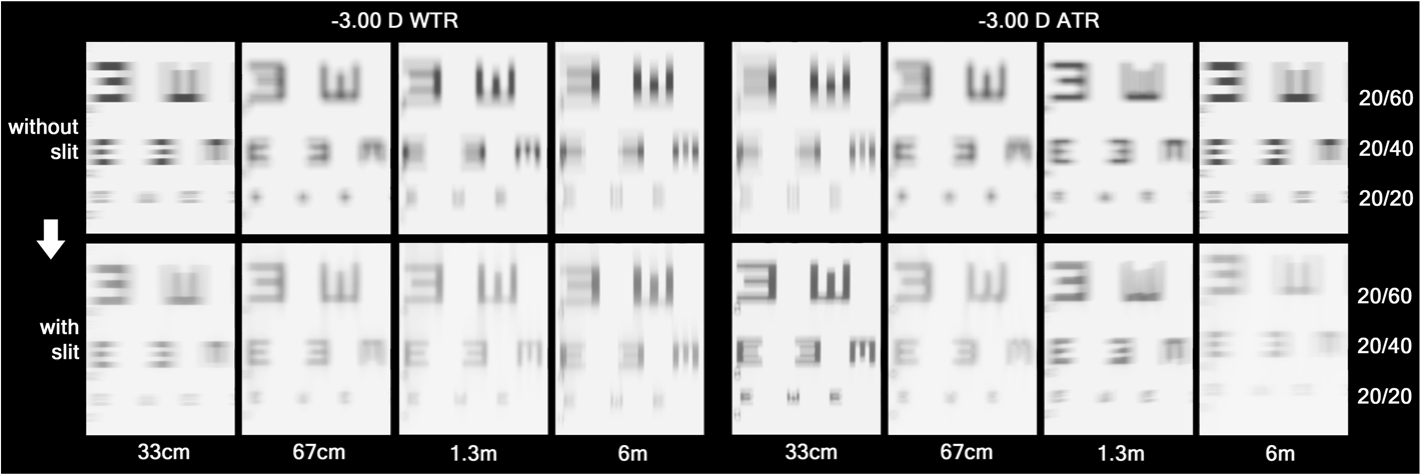
Simulated vision chart seen through the refractive statuses of − 3.00 D WTR and ATR simple myopic astigmatism (COAS vision simulation program). Without the slit, both groups could see best at 67 cm distance (at which the circle of least confusion coincides on the retina). With the slit, the distance at which the vertical focal lines lie on the retina showed better vision than before (6 m of the − 3.00 D WTR astigmatism and 33 cm of the − 3.00 D ATR astigmatism). Note that the vision at 33 cm is comparable to that at 67 cm of the − 1.50 D ATR astigmatism (Fig. 3)

VA chart simulation was done at each refractive status, with and without the slit (Figs. 3 and 4). Only the optotypes of 20/60 or smaller were shown at the figures because larger optotypes were legible in all groups. With the slit in front, the WTR astigmatism groups could see better at distance than at near, while the ATR astigmatism groups had the opposite results. Even optotypes of 20/20 at near were legible with the slit in place in the ATR astigmatism groups. When the horizontal slit was placed in front, best vision was achieved at the 6 m plane in − 1.50 D WTR and − 3.00 D WTR astigmatism, at 67 cm in − 1.50 D ATR astigmatism, and at 33 cm in − 3.00 D ATR astigmatism, respectively.

## Discussion

**Fig. 5:**
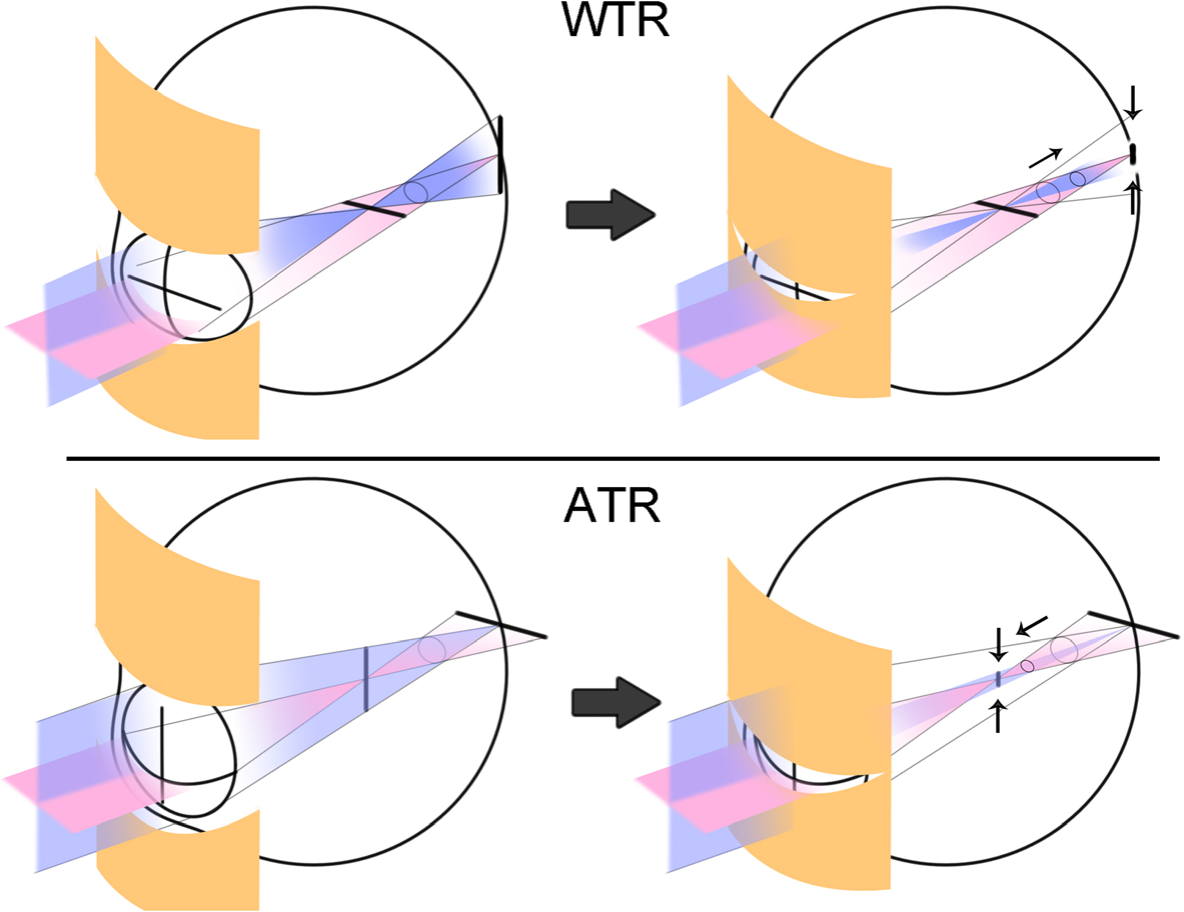
Schematic illustration of the effects of the eyelid on astigmatic focal lines. As the eyelid fissure size becomes smaller than the diameter of the entrance pupil, a slit-like effect occurs and eventually shortens the vertical focal lines. In simple ATR astigmatism, the focus (circle of least confusion) moves to the nearer plane; in WTR astigmatism, the focus moves to the farther plane

This study revealed that, in simple myopic astigmatism, squinting induces a focus shift in the opposite directions in WTR versus ATR astigmatism. This pseudoaccommodation effect can cause an overestimation of near VA when squinting is not prohibited. Figure 5 shows an easily-understood schematic diagram of the slit effect on vertical blur. One of the two focal lines (vertical and horizontal) in simple myopic astigmatism is located *on* the retina (distance), while the other is located *in front of* the retina (near). In WTR astigmatism, the vertical focal line is located *on* the retina (distance), while, in ATR astigmatism, it is located *in front of* the retina (near). Because the eyelids act as a slit, light rays passing the vertical meridian of cornea and lens are obstructed, and, thus, vertical blur decreases as the vertical focal line shortens. The shortening of the focal line decreases point spread function in exchange for a decreased amount of light (contrast). It follows then that squinting may improve distance vision in WTR astigmatism and near vision in ATR astigmatism, respectively. Anyone can easily experience an improvement in blurred vision by squinting, if positive cylindrical lenses were put in front of one’s own eyes inducing WTR or ATR astigmatism and squinting was attempted.

The circle of least confusion shifts from the middle of the two focal lines to a more distant point (closer to the retina) in WTR astigmatism and to more near point (far from the retina) in ATR astigmatism (Fig. 5). The hyperopic shift of focus term of the Seidel aberrations coefficient in the WTR astigmatism and myopic shift of such in the ATR astigmatism that occurred in the present study corroborated this point. On the other hand, the spherical equivalent did not show a similar change to that of the focus term. This may be because the spherical equivalent is located at the arithmetical mean point of the two focal lines and the slit only changes the length of the vertical focal lines but not the position of the focal lines themselves. Thus, the spherical equivalent does not change, unlike the circle of least confusion.

**Fig. 6:**
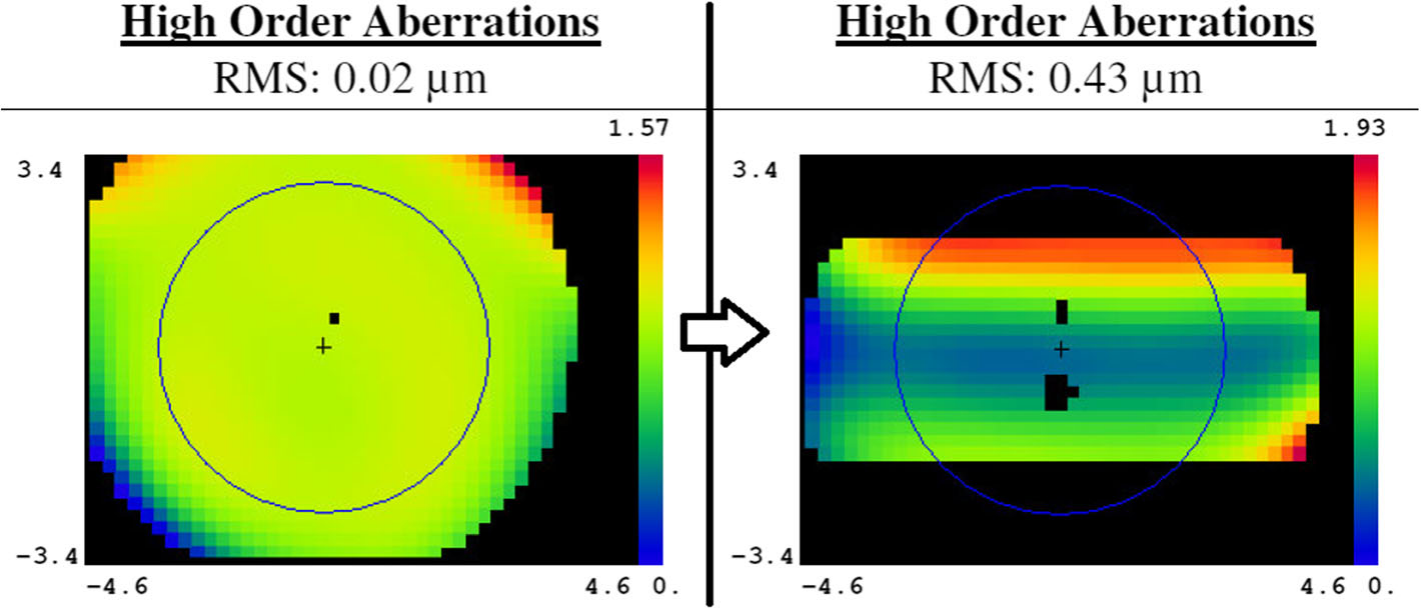
High-order aberrations change with the horizontal slit on the emmetropia, showing aberration caused by diffraction at the slit margin

In the present study, placement of a horizontal slit induced an increase in WTR astigmatism (i.e., it induced an increase of astigmatism in the emmetropia and WTR astigmatism groups, while a decrease of astigmatism in the ATR astigmatism groups). However, such a model by itself cannot explain the observed change in astigmatism. If the superior and inferior vertical meridians are just subtracted, the amount of astigmatism should be the same or decreased due to the decreased difference in the vertical and horizontal meridian components. However, in the WTR astigmatism groups, astigmatism was increased. This is probably due to the diffraction effect of the slit margin. If a person squints their eyes and gazes at a light source, they will generally notice a vertical straylight. This phenomenon is due to the diffraction at the eyelid margin. Grey and Yap’s observation of increased WTR astigmatism when squinting corresponds well with our finding [28]. All of the groups with the slit showed the shift from ATR to WTR astigmatism. Even in the emmetropia group, in which no astigmatism was present before placing the slit, WTR astigmatism and horizontal higher-order aberrations were induced by the slit (Table 1; Fig. 6). Furthermore, astigmatism was decreased to a greater degree than expected in the ATR astigmatism groups. ATR astigmatism with squinting, therefore, may lead to better near vision, not only due to a decrease in vertical blur but also by a decrease in the astigmatism itself. The exact mechanism and amount of WTR astigmatism-like aberration induced by diffraction should be elucidated by further study. In real human eyes, factors like the concave lens effect of the tear meniscus, the eyelid fissure’s curved shape, and eyelid pressure-induced changes in corneal astigmatism should also be considered. It is a limitation of this study that only a pure optical effect of squinting was taken into account.

This study revealed that, together with eyelid fissure, myopic astigmatism ensures a greater range of pseudoaccommodation than previously expected. Considering the pseudoaccommodation range of WTR and ATR astigmatism, the target refraction of cataract surgery could be adjusted to maximize patient satisfaction when residual astigmatism is expected to be left behind postoperatively. We suggest that target refraction of a more myopic nature is better than targeting emmetropia wherein the distance vision of WTR astigmatism and near vision of ATR astigmatism will lose benefit. This coincides with the result of Sawusch and Guyton’s study, which demonstrated that the optimal combination occurs when the negative sphere is 0.25 D greater in magnitude than the positive cylinder; e.g., − 0.50 D = + 0.75 D × 90 [36]. However, their study did not consider different astigmatism orientations and the effects of eyelid fissure. Residual simple myopic ATR astigmatism contributes to near vision, and this could explain better the near VA of ATR astigmatism observed in many studies [3–7]. It is interesting to find out that, when comparing the VAs of − 1.50 D ATR and − 3.00 D ATR astigmatism at 33 cm wherein the vertical focal line of the − 3.00 ATR astigmatism coincides on the retina, − 3.00 D ATR astigmatism showed comparable VA and more dense contrast than did − 1.50 D ATR astigmatism with the placement of a horizontal slit. Even having more astigmatism than − 1.50 D ATR astigmatism, the model with − 3.00 D ATR astigmatism could see better at 33 cm with squinting.

Only Seidel aberration terms were used in the compariosn of the present study, instead of Zernike aberration terms. The Zernike polynomials are orthogonal on the unit circle. Since we used a slit to occlude part of the circle, Zernike polynomials could be inaccurate if applied to analyze the wavefront map. Additionally, Zernike polynomials can only be translated into Seidel aberrations if the higher-order aberrations are small enough to be neglected. In this study, the changes in aberrations were dispersed in various polynomials and were not easily understood intuitively.

## Conclusions

The presence of an eyelid fissure smaller than the pupil decreases vertical blurring and moves the focus in opposite directions in WTR and ATR astigmatism, respectively. The diffraction effects of the eyelid could induce a WTR-like astigmatism change. Eyelid squinting improves distance vision in WTR and near vision in ATR astigmatism in pseudophakic eyes. These pseudoaccommodation effects of the eyelids on ATR astigmatism may cause overestimation of near VA when squinting is not prohibited.

## Data Availability

The datasets during and/or analysed during the current study available from the corresponding author on reasonable request.

## Abbreviations

ATR: Against-the-rule
COAS: Complete Ophthalmic Analysis System
D: Diopters
VA: Visual acuity
WTR: With-the-rule
